# Let's jump in: A phylogenetic study of the great basin springfishes and poolfishes, *Crenichthys* and *Empetrichthys* (Cyprinodontiformes: Goodeidae)

**DOI:** 10.1371/journal.pone.0185425

**Published:** 2017-10-27

**Authors:** D. Cooper Campbell, Kyle R. Piller

**Affiliations:** Southeastern Louisiana University, Dept. of Biological Sciences, Hammond,United States of America; National Cheng Kung University, TAIWAN

## Abstract

North America’s Great Basin has long been of interest to biologists due to its high level of organismal endemicity throughout its endorheic watersheds. One example of such a group is the subfamily Empetricthyinae. In this paper, we analyzed the relationships of the Empetrichtyinae and assessed the validity of the subspecies designations given by Williams and Wilde within the group using concatenated phylogenetic tree estimation and species tree estimation. Samples from 19 populations were included covering the entire distribution of the three extant species of Empetricthyinae–*Crenichthys nevadae*, *Crenichthys baileyi* and *Empetricthys latos*. Three nuclear introns (S8 intron 4, S7 intron 1, and P0 intron 1) and one mitochondrial gene (Cyt*b*) were sequenced for phylogenetic analysis. Using these sequences, we generated two separate hypotheses of the evolutionary relationships of Empetrichtyinae- one based on the mitochondrial data and one based on the nuclear data using Bayesian phylogenetics. Haplotype networks were also generated to look at the relationships of the populations within Empetrichthyinae. After comparing the two phylogenetic hypotheses, species trees were generated using *BEAST with the nuclear data to further test the validity of the subspecies within Empetrichthyinae. The mitochondrial analyses supported four lineages within *C*. *baileyi* and 2 within *C*. *nevadae*. The concatenated nuclear tree was more conserved, supporting one clade and an unresolved polytomy in both species. The species tree analysis supported the presence of two species within both *C*. *baileyi* and *C*. *nevadae*. Based on the results of these analyses, the subspecies designations of Williams and Wilde are not valid, rather a conservative approach suggests there are two species within *C*. *nevadae* and two species within *C*. *baileyi*. No structure was found for *E*. *latos* or the populations of Empetricthyinae. This study represents one of many demonstrating the invalidity of subspecies and their detriment to species identification, conservation, and understanding.

## Introduction

The recognition of subspecies has been hotly debated [1][2][3][4][5] among researchers since the development of the evolutionary species concept, which defines a species as “a single lineage of ancestor-descendent populations of organisms which maintains its identity from other such lineages and which have their own evolutionary tendencies and historical fate” [6]. As a result, there are multiple described issues with the use of subspecies [1][2][7][8][9]. Philosophically, they represent an error of over-reductionism, and do not represent true species boundaries based on species as individuals [2][3][10]. As a result, the subspecies concept is poorly defined throughout the literature, which further obfuscates species relationships within groups of organisms and confounds scientists [5][11]. For example, subspecies have been described as potential incipient species [12], populations that are geographically separate but phenotypically similar [13], or as species that show differences across a cline [7][14]. Mayr [1] later described subspecies as tools for taxonomists to use when organizing species within collections rather than actual evolutionary units. Because of these issues, subspecies as a taxonomic unit were quickly recognized as poorly defined and having no real lower limit [7]. Furthermore, they compound problems because they cannot predict that further speciation will or will not continue within these groups [14].

The confusion associated with the designation and recognition of subspecies also can confound conservation efforts and lead to the introduction of incorrect or unnecessary policy, resulting in a reduction in conservation efficiency [3][15][16]. As a result, many biologists utilize the evolutionary species concept to recognize species as the unit of conservation [14][17][18]. This application has proven useful for identifying units of conservation as well as the discovery of previously unrecognized cryptic species [19][20][21][22]. Taxonomic diversity that is reflective of the evolutionary history can allow resource managers to more accurately manage and protect jeopardized species.

**Fig 1:**
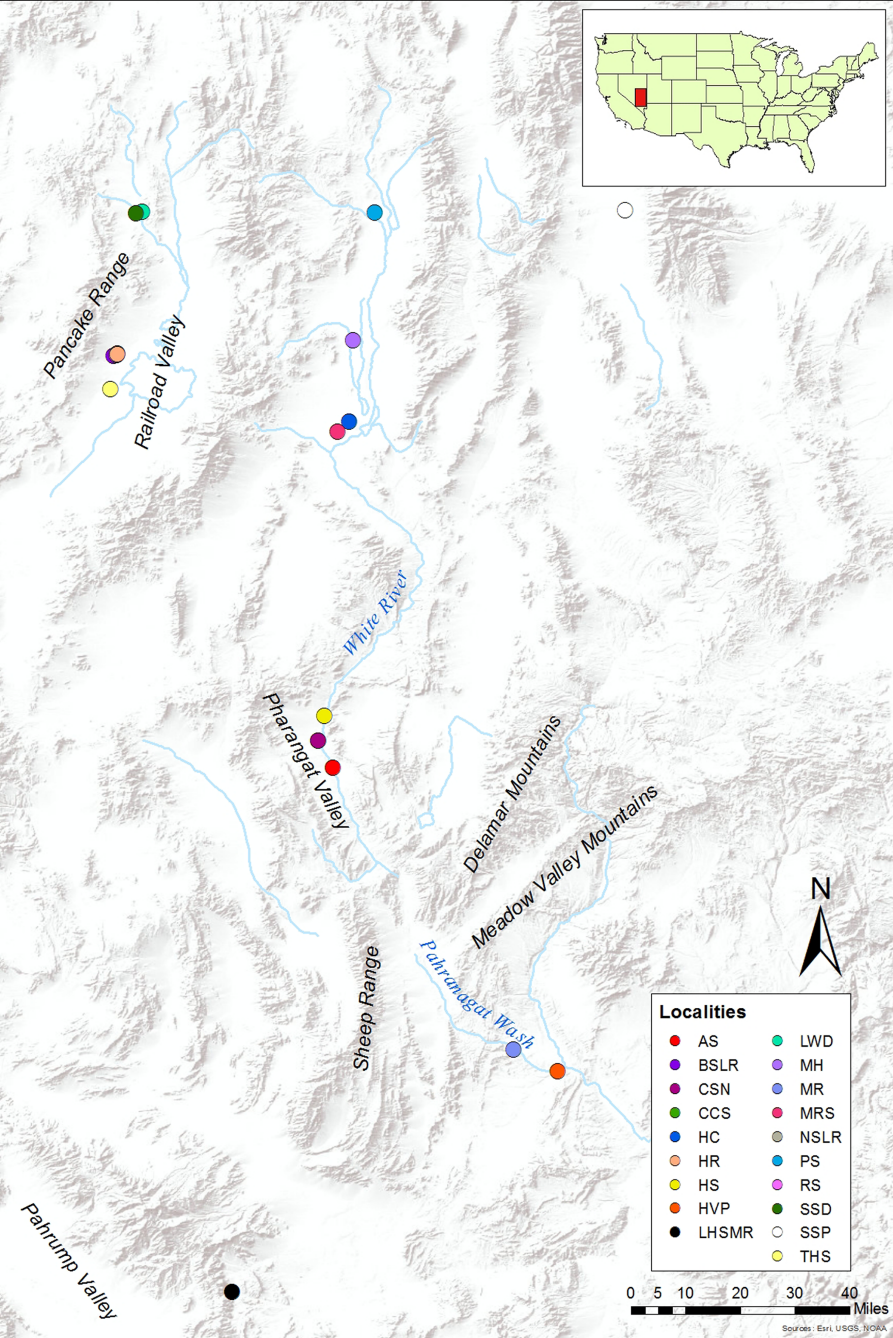
Distribution Map. Map of the great basin in nevada showing the locations of the samples collected from each population as well as significant geologic features separating populations. Abbreviations match the locality labels presented on Table 1. Map used: World Terrain Base; data sources: Esri, USGS, NOAA; Used with permission from ESRI License Agreement E204 08/10/2017, original copyright June 2009 [27].

Unfortunately, the conservation status of many groups of organisms is plagued by inconsistent and unwarranted use of subspecies. The genus *Crenichthys* (Cyprinodontiformes: Goodeidae) is a narrowly restricted group of fishes endemic to the Great Basin of the western United States. *Crenichthys* and its sister genus *Empetrichthys* represent the two genera within the subfamily Empetrichthyinae (Goodeidae). *Crenichthys* contains two species, *C*. *baileyi* and *C*. *nevadae*, and multiple subspecies. *Crenichthys baileyi* is distributed across the pluvial White River of southeastern Nevada, a disjunct collection of streams that ultimately become the Pahranagat Wash before flowing into Lake Mead along the Nevada-Arizona border (Fig 1). Williams and Wilde [23] described five subspecies of *C*. *baileyi* [24], including *C*. *b*. *albivalis*, *C*. *b*. *baileyi*, *C*. *b*. *grandis*, *C*. *b*. *moapae*, and *C*. *b*. *thermophilus*, based on a spectrum of overlapping morphologic characters and subjective pigment differences, each of which are separated across individual steams and pools. Populations of *C*. *nevadae* are morphologically less variable from each other and are distributed within springs throughout Railroad Valley, west of the White River in central Nevada. *Empetrichthys* consists of a single extant species, *E*. *latos*, extirpated from its native range, which only survives in an area outside of is former native range in Shoshone stock pond, Corn Creek, and Lake Harriet. Additional taxa within *Empetrichthys* have gone extinct including *E*. *latos concavus*, *E*. *latos pahrump*, and *E*. *merriami* [25][26].

As a whole, the subfamily Empetrichthyinae is an imperiled group with two species and multiple subspecies listed at the federal level by the U.S. Fish and Wildlife Service [28]. Currently, *C*. *nevadae* is listed as threatened throughout its range at the federal level. Within *C*. *baileyi*, *C*. *b*. *baileyi* and *C*. *b*. *grandis* are currently listed as endangered federally, while the other subspecies are unlisted at the federal level but are listed at the state level [29]. *Empetrichthys latos* is listed as endangered at the federal level [30][31][32]. The entire group has a relatively restricted range, relative to its more diverse sister subfamily Goodeiane, which is widespread across central Mexico [33]. Little taxonomic work has been done with the species and populations within the Empetrichthyinae despite their endangered status. Significant anthropogenic effects including the introduction of invasive species and destruction of habitat [23][34][35][36] have been the main causes for their decline and continue to jeopardize the long-term persistence of the populations within the subfamily.

The recognition of subspecies has confounded scientific study and masked taxonomic diversity within many species, thereby impacting our understanding of evolutionary relationships and our ability to inform conservation efforts to protect natural diversity [3][15][16]. With this in mind, the subfamily Empetrichthyinae represents a perfect opportunity to understand evolutionary relationships and to test taxonomic boundaries within this imperiled group of fishes. The objectives of this study were (1) to obtain phylogenetic hypotheses of the relationships of all of the extant species and subspecies of Empetrichthyinae across their entire range using both mtDNA (cytochrome b) and nDNA (S7 ribosomal intron-1, S8 intron-4, and P0 intron-1) sequences and to (2) compare the obtained relationships with the currently accepted taxonomic boundaries within the subfamily. Using two separately inferred trees obtained with mtDNA and nDNA provides two independent hypotheses of relationships within Empetrichthyinae. Phylogenetic and species delimitation approaches were also conducted and provide a new picture of the relationships and diversity within the group. The results from this study will inform future conservation efforts, and fill in the gaps on the genetics and relationships of Empetrichthyinae, while encouraging a re-evaluation of their taxonomy.

## Methods

### Study area

For this study, fin clips preserved in 95–100% ethanol were collected by personnel from the Nevada Department of Wildlife, representing nearly all extant population of Empetrichthyinae (19 different localities, geographically separate from one another) (Table 1). The tissue samples used in this study were collected by biologists of the Nevada Department of Wildlife and provided to KRP. The animal care protocols employed in this study fall within the approved IACUC#0002 protocol approved by Southeastern Louisiana University. Specimens of *C*. *nevadae* were collected from seven localities including Big Spring Loches Ranch, North Spring Loches Ranch, Little Warm Duckwater, School Spring Duckwater, Reynolds Spring, Haycorral Loches Ranch, and Terrace Hot Spring. Samples of *C*. *baileyi* were obtained from ten localities representing each subspecies including *C*. *b*. *albivalis* (Preston Spring), *C*. *b*. *baileyi* (Ash Spring), *C*. *b*. *grandis* (Crystal Springs South, Crystal Springs North, Hiko Spring), *C*. *b*. *moapae* (Warm Springs, Hidden Valley Pond), and *C*. *b*. *thermophilus* (Moon River Hot Springs, Moorman Hot Springs, Hot Creek). Lastly, samples of *Empetrichthys latos* were provided from two stock ponds (Shoshone Stock Pond and Lake Harriet) as they have been extirpated from their natural habitat [25][37] and new populations established. Genomic DNA from the fin clips were extracted using the the DNeasy Kit (Qiagen, Inc.) following the manufacturers recommendations.

**Table 1:** Sample information. Taxon, population ID label, collection site, latitude, longitude, and number of individuals sequenced for each gene.

Taxon	Taxon label	Locality Label	Collection Site	Latitude	Longitude	cytb n =	P0 n =	S7 n =	S8 n =
*Crenichthys nevadae*	*Cn*	BSLR	Big Springs Loches Ranch	38.55	-115.77	4	4	4	2
		NSLR	North Springs Loches Ranch	38.56	-115.77	3	5	5	3
		LWD	Little Warm Duckwater	38.93	-115.70	4	5	5	5
		SSD	School Spring Duckwater	38.93	-115.72	4	5	5	5
		RS	Reynolds Spring	38.55	-115.77	4	5	5	5
		HR	Haycorral Loches Ranch	38.56	-115.76	4	5	5	5
		THS	Terrace Hot Spring	38.46	-115.78	5	4	5	5
*C*. *baileyi thermophilus*	*Cbt*	MRHS	Moon River Hot Springs	38.38	-115.15	4	4	5	3
		MHS	Moorman Hot Springs	38.35	-115.18	4	5	5	5
		HC	Hot Creek	38.59	-115.14	4	4	5	4
*C*. *b*. *moapae*	*Cbm*	MR	Muddy River	36.71	-114.71	5	5	5	5
		HVP	Hidden Valley Pond	36.65	-114.60	5	5	5	5
*C*. *b*. *grandis*	*Cbg*	CSS	Crystal Springs South	37.53	-115.23	4	5	5	3
		CSN	Crystal Springs North	37.53	-115.23	4	6	6	4
		HS	Hiko Spring	37.6	-115.22	5	5	5	4
*C*. *b*. *baileyi*	*Cbb*	AS	Ash Spring	37.46	-115.19	5	4	5	5
*C*. *b*. *albivalis*	*Cba*	PS	Preston Spring	38.93	-115.08	3	4	4	3
*Empetrichthys latos*	*El*	SSP	Shoshone Stock Pond	38.94	-114.42	5	3	5	5
		LHSMR	Lake Harriet, Spring Mount. Ranch	36.07	-115.46	3	5	5	4

### Sequence data

One mitochondrial gene and three nuclear introns were included in this study. For the mitochondrial gene cytochrome *b*, at least 3 individuals from each population were sequenced for a total of 79 specimens amplified. Amplification protocol for cyt*b* utilized primers described by Schmidt and Gold [38] and proceeded as follows: initial denaturation at 94°C for 2 min, 27 cycles of 95°C for 45 s, 54°C for 30 s, and 72°C for 2 min, and a final extension at 72°C for 10 min. For each nuclear intron (S7 intron-1, S8 intron-4, & P0 intron-1), at least 3 individuals from every population were sequenced, resulting in a total of 94, 82, and 90 sequences respectively for each intron. For S7 intron-1, primers described by Chow and Hazama [39] were used for amplification. The PCR protocol for S7 intron-1 was as follows: initial denaturation at 94°C for 4 min, 30 cycles of 95°C for 45 s, 60°C for 45 s, and 72°C for 1 min, and a final extension at 72°C for 4 min. For S8 intron-4 and P0 intron-1, primers described by Chow and Yanagimoto [40] were used. Seven other nuclear introns were amplified, however, none of these introns were phylogenetically informative at the species level so they were excluded from this study. For S8 intron-4 and P0 intron-1, touchdown protocols were used [41]. For S8 intron-4, the protocol was initial denaturation at 94°C for 3 min, 32 cycles of 94°C for 30 s, an initial temperature of 70°C for 30 s for the first cycle that reduced by 1°C each successive cycle until remaining constant at 61°C, followed by 72°C for 1 min, and a final extension at 72°C for 7 min. For P0 intron-1, the protocol was initial denaturation at 94°C for 3 min, 32 cycles of 94°C for 30 s, an initial temperature of 68°C for 30 s for the first cycle that reduced by 1°C each successive cycle until remaining constant at 58°C, followed by 72°C for 1 min, and a final extension at 72°C for 7 min. All reactions for all loci were performed in solution with a total volume of 25 uL reactions in 0.5 mL tubes containing 1 uL template DNA, 19 uL water, 0.75 uL MgCl, 2.5 uL 10X buffer, 0.5 uL dNTP, 0.5 uL of forward and reverse primer, and 0.25 uL of taq polymerase. Upon successful visualization of product on a 0.8% agarose gel, samples were sequenced by Genewiz [42]. Forward and reverse sequences received were then aligned using Geneious v9.1.3 software before submitting the sequences to Genbank (MF578383-MF578739).

### Phylogenetic analysis

Genetic distance for each locus (both mitochondrial and nuclear) was calculated using pairwise uncorrected *p* distance in MEGA v7.0.18 [43]. Phylogenetic hypotheses were developed for cyt*b* and each individual intron using Bayesian Inference (BI) analysis with MrBayes v3.2.6 [44] within the CIPRES portal [45]. The three nuclear introns used also were concatenated and analyzed to produce a tree. PartitionFinder v1.1.1 [46] was used to determine the best fitting codon partitioning scheme (for cyt*b*) and nucleotide substitution models for the nuclear introns and mitochondrial data using Bayesian information criterion scoring. Markov Chain Monte Carlo (MCMC) repetitions were run for 15,000,000 generations with trees sampled every 6,000 generations and a burnin fraction of 25% for analysis of cyt*b*. For the analysis of each individual intron and the concatenated nuclear dataset, Markov Chain Monte Carlo (MCMC) repetitions were run for 15,000,000 generations with every 10,000 trees sampled with a burnin fraction of 25%. Burnin fractions were determined by observing the log likelihood scores plotted in Tracer v1.6. The default number of runs (n = 2) was used for each analysis. All ESS values obtained were greater than 200 with average standard deviations of split frequencies less than 1 and potential scale reduction factors equaling one, all indicating that the runs had reached convergence. Sequence data from three species of goodeids, sequenced in this study or obtained from Genbank (AF510824.1 & KC778798.1), from the sister subfamily Goodeinae (*Characodon audax*, *Xenotoca eiseni*, *Ilyodon whitei*) were included along with an outgroup member of Profundulidae (*Profundulus candalarius*) sequenced in this study to root the trees. The trees obtained and posterior probabilities (BPP) were visualized in Figtree v.1.8.3.

## Haplotype networks

Haplotype networks based on cyt*b* were created for each species currently recognized within Empetrichthyinae using PopART v1.7 to develop 50% majority rule median joining networks [47]. The cyt*b* sequences used for the haplotype network analysis were trimmed to the shortest sequence (1086 bp) to avoid overestimating unique haplotypes. A network was first created with all the collected sequences within the subfamily of Empetrichthyinae to observe the number of substitution differences between haplotypes and then separated to create networks for each species and their respective localities. Separation of these groups and within each haplotype network were supported by TCS v1.21 [48] analysis using a 95% cutoff criterion. All parameters within PopART and TCS were left at default values.

### Species tree analysis

Species trees were constructed using *BEAST [49][50] through the CIPRES portal using the nuclear introns P0, S7, and S8. All individuals were used in the analysis with species designations based on the *cytb* mitochondrial lineages discovered herein. A clock model test was executed for each locus using a likelihood ratio test in PAUP* v.4.0a152 [51]. For the speciation prior, a Yule Process parameter was used. The analysis was run for 1,000,000,000 generations sampling every 200,000 for a total of 5,000 trees with a 10% burnin of 500 trees. The log files were analyzed in TRACER v.1.6 to check for convergence and ESS values. All ESS values obtained were greater than 1,500. The remaining 4,500 trees were analyzed in TreeAnnotator v.1.8.4 to obtain a maximum clade credibility tree. The obtained tree and posterior probabilities (BPP) were visualized in Figtree v.1.8.3. Trees with species designations based on the nuclear lineages and the morphological hypothesis of Williams and Wilde [23] were also run with the same parameters.

## Results

### Sequence alignment and model selection

Sequences were aligned separately and trimmed to make sequences identical in length. The final length for *cytb* was 1024 bp, for S7 was 573 bp, for S8 was 498 bp, and for P0 was 281 bp. The best-fitting partition schemes and models of substitution for phylogenetic analysis for each gene were as follows: *cytb* by codon partition is GTR+I+G, HKY+I, and GTR+G, S7 is GTR+G, S8 is HKY+G, and P0 is HKY+I. Substitution models were adjusted to suit options available in *BEAST for analysis using jModelTest [52] for nuclear introns (JC for P0, HKY for S7 and S8).

### Phylogenetic trees

**Fig 2:**
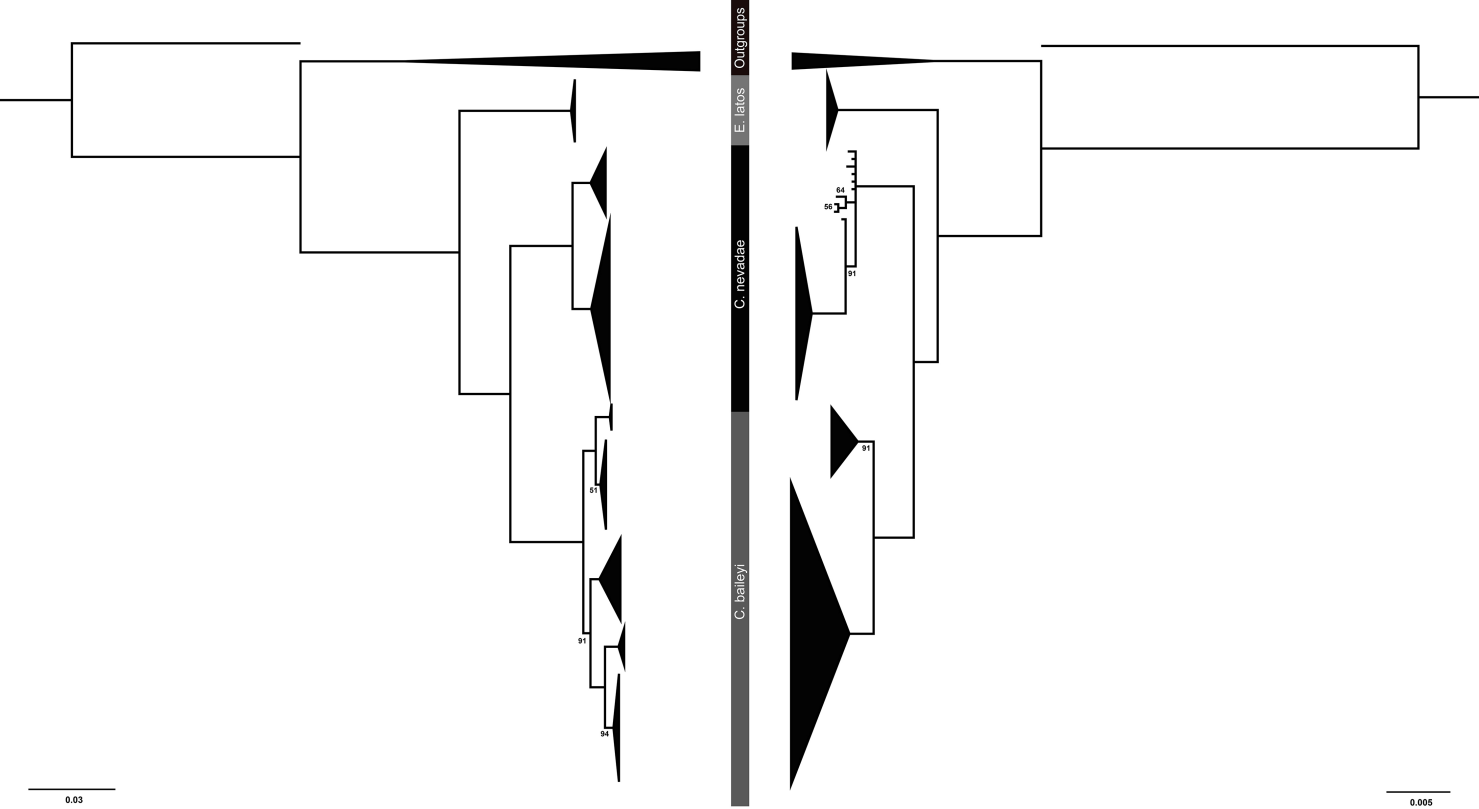
MtDNA and nDNA phylogenies. Fifty-percent majority rule consensus trees of a codon partitioned mixed model Bayesian analysis of cytochrome b (left) and fifty-percent majority rule consensus trees of a gene partitioned mixed model Bayesian analysis of concatenated nuclear introns P0 intron 1, S7 intron 1, and S8 intron 4 (right). Taxonomic labels follow Williams and Wilde, 1981. All posterior probabilities greater than 95 unless presented.

Both the mitochondrial dataset and the concatenated nuclear dataset supported the monophyly of Empetrichthyinae (BPP = 100) (Fig 2). These analyses recovered a clade containing *Crenichthys* with *C*. *nevadae* sister to *C*. *baileyi* (BPP ≥ 95). This clade, in turn, was sister to *E*. *latos* (BPP = 100), however, relationships of the populations within each species varied between the mtDNA and nDNA trees. In general, the mitochondrial tree had more population structure than the concatenated nuclear tree, which is to be expected [53].

**Fig 3:**
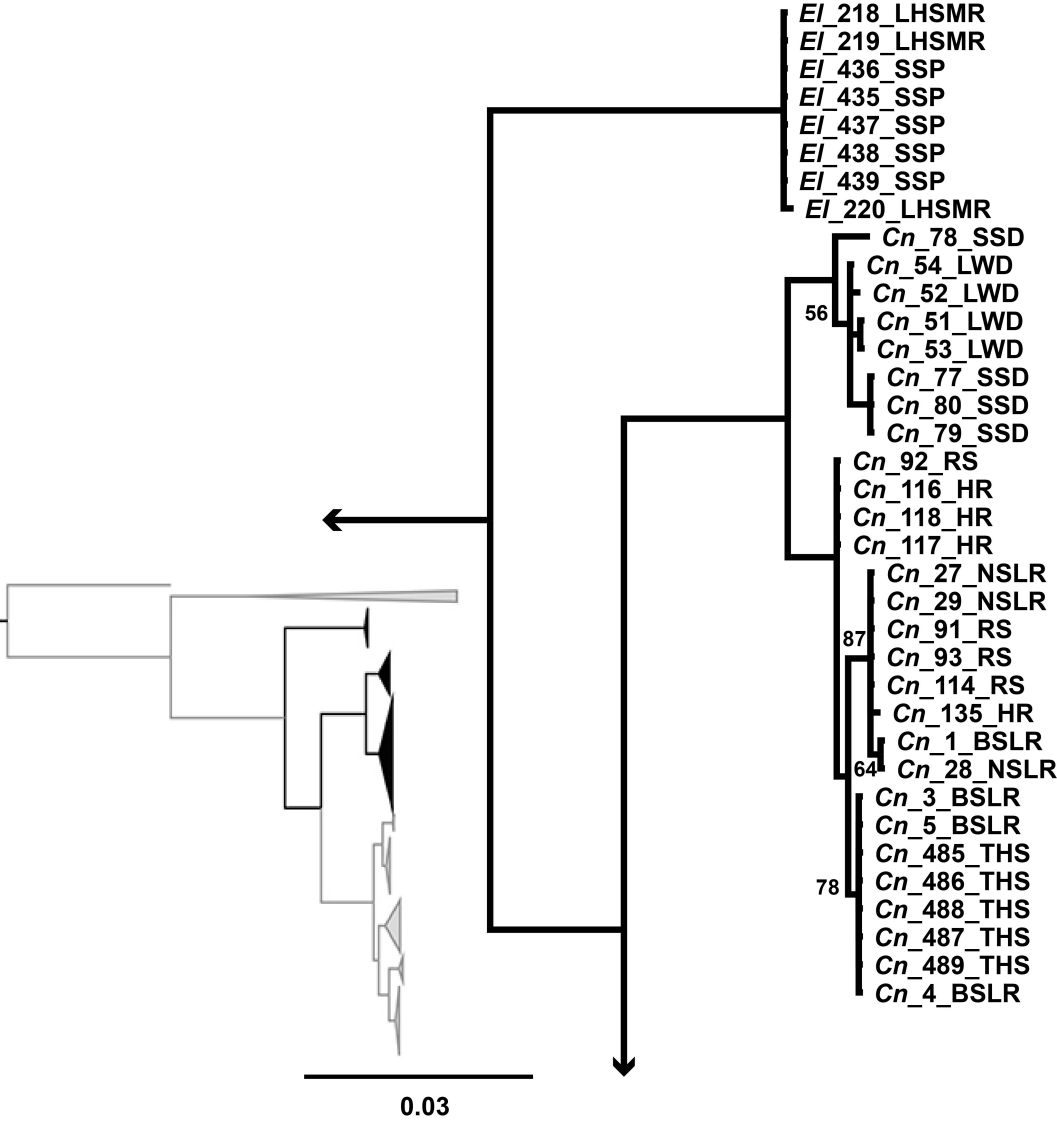
MtDNA subtree of *C*. *nevadae* and *E*. *latos*. Mitochondrial subtree depicting the northern and southern clades of *Crenichthys nevadae* as well as the clade containing *Empetrichthys latos*. All posterior probabilities greater than 95% unless specified on the tree.

Within the mitochondrial tree, the *C*. *nevadae* clade contained two major lineages with specimens from the southern portion of the range including Big Springs Loches Ranch, North Springs Loches Ranch, Reynolds Spring, Haycorral Loches Ranch, and Terrace Hot Spring forming one clade (BPP ≥ 95), hereafter called the southern clade, and the other specimens from the northern portion of the range including Little Warm Duckwater and School Spring Duckwater forming a sister clade (BPP ≥ 95), hereafter called the northern clade (Fig 3). Although there was some structure within both the northern and southern clades, the analysis did not separate any of the populations from one another within these clades, so many haplotypes were shared across localities but not between the two clades.

**Fig 4:**
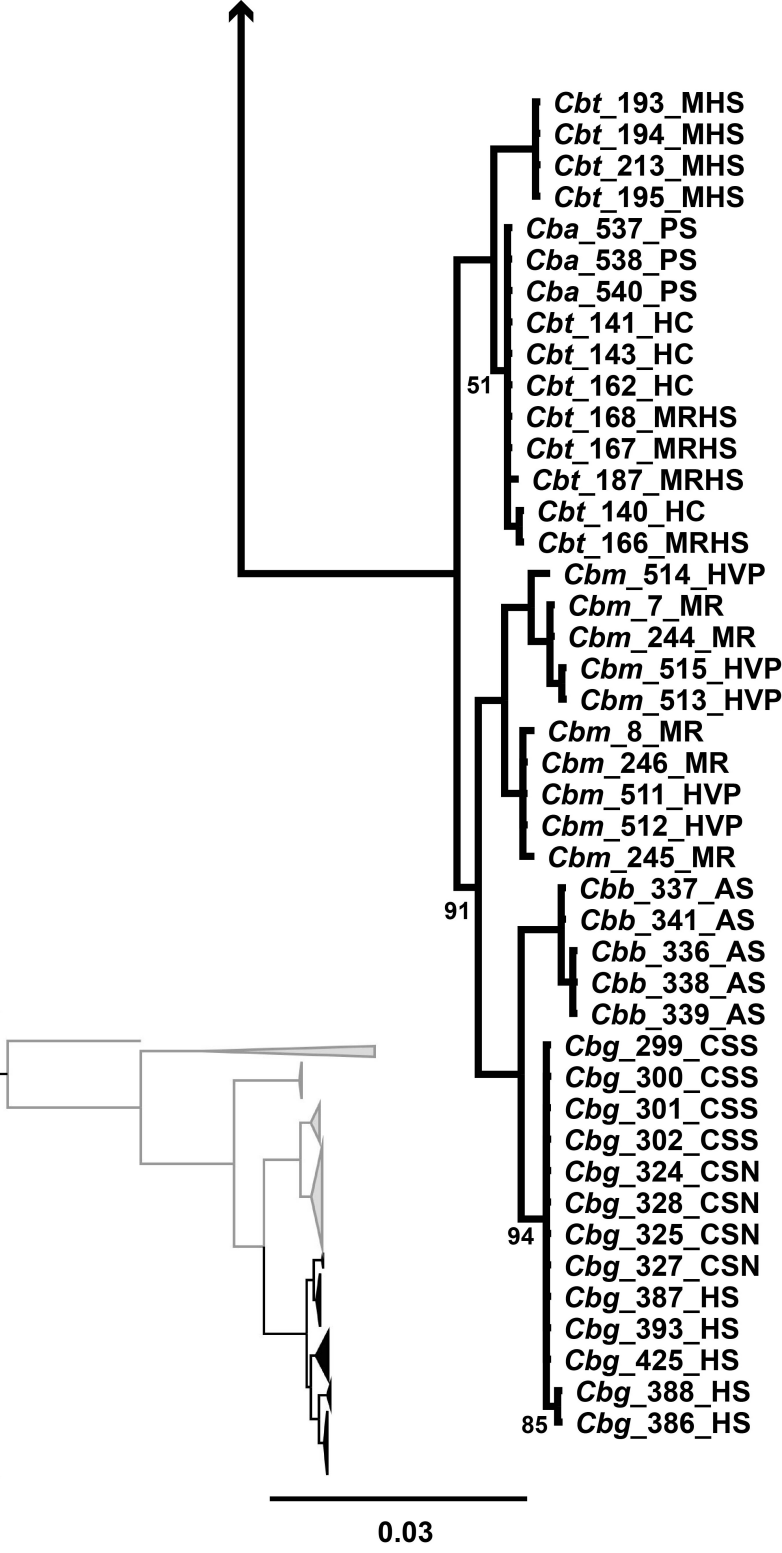
MtDNA subtree of *C*. *baileyi*. Mitochondrial subtree depicting the subspecies relationships of *Crenichthys baileyi*. All posterior probabilities greater than 95% unless specified on the tree.

Within the *C*. *baileyi* clade in the mitochondrial tree, only four of the five recognized subspecies formed clades (Fig 4). A clade containing all the individuals of *C*. *b*. *themophilus* and *C*. *b*. *albivalis* was recovered, and was sister to the other members of *C*. *baileyi* (BPP = 100). Within this clade, samples of *C*. *b*. *thermophilus* from Moorman Hot Springs formed a monophyletic clade (BPP = 100) while samples from Moon River Hot Springs, Hot Creek, and samples of *C*. *b*. *albivalis* from Preston Springs formed a separate clade with low posterior probability (BPP = 50) and no population structure. The other three subspecies *C*. *b*. *moapae*, *C*. *b*. *baileyi*, and *C*. *b*. *grandis* formed monophyletic clades (BPP ≥ 90). Within this clade, each subspecies formed monophyletic clades (BPP ≥ 94) with *C*. *b*. *moapae* sister to a clade containing *C*. *b*. *grandis* and *C*. *b*. *baileyi* sister to one another. No population structure was present within these clades.

**Fig 5:**
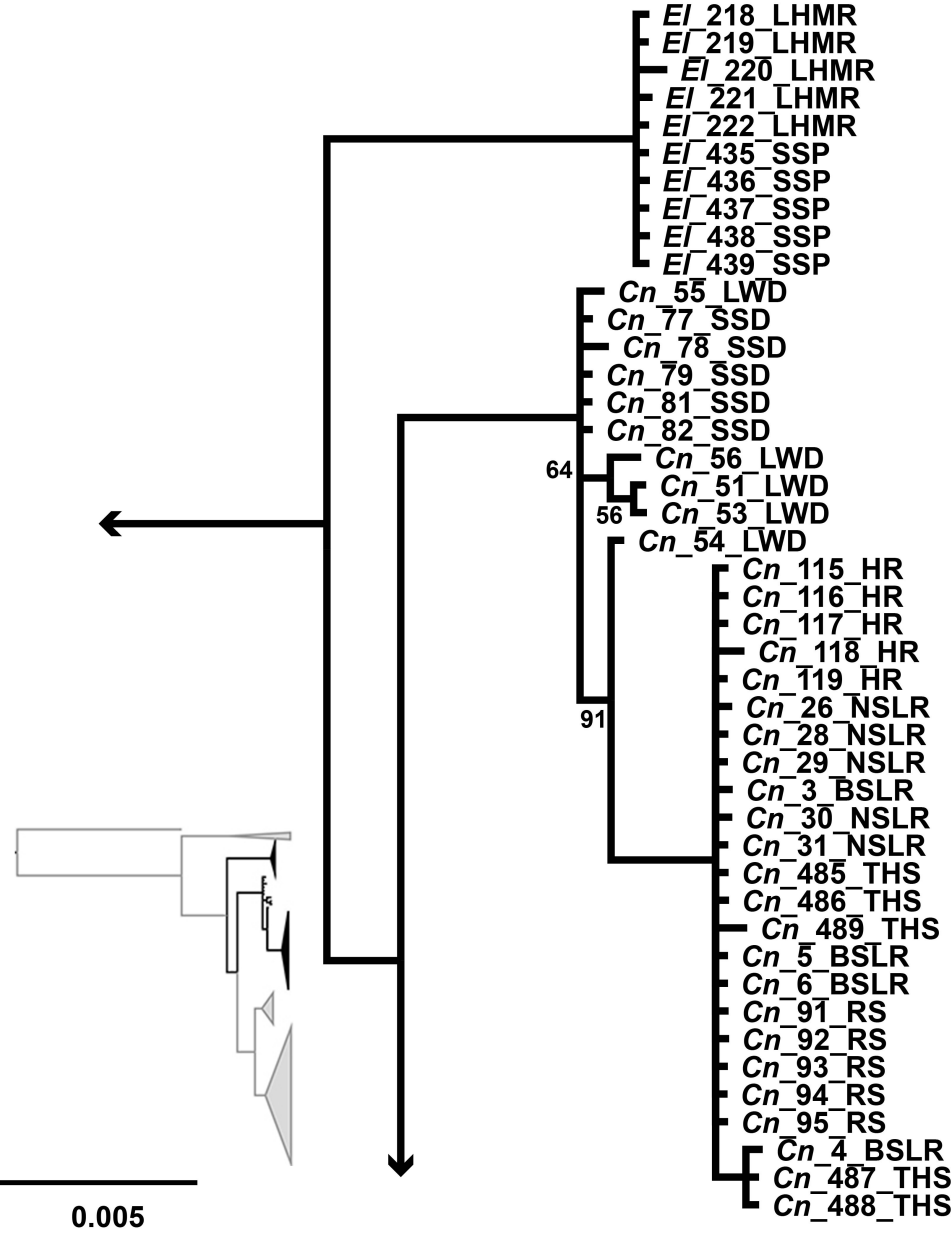
NDNA subtree of *C*. *nevadae*. Nuclear subtree depicting the relationships of *Crenichthys nevadae*, here only the Southern clade has been retained compared to the mitochondrial tree. All posterior probabilities greater than 95% unless specified on the tree.

For the nuclear intron data for *C*. *nevadae*, the southern clade was recovered again and once again formed a clade (BPP = 100) that was sister to specimens from the northern clade (BPP = 100), however, the samples from the northern clade formed an unresolved polytomy on the tree rather than a clade (Fig 5). No population structure was present. Individual genes trees are presented in supplemental figures (S1 Fig, S2 Fig and S3 Fig).

**Fig 6:**
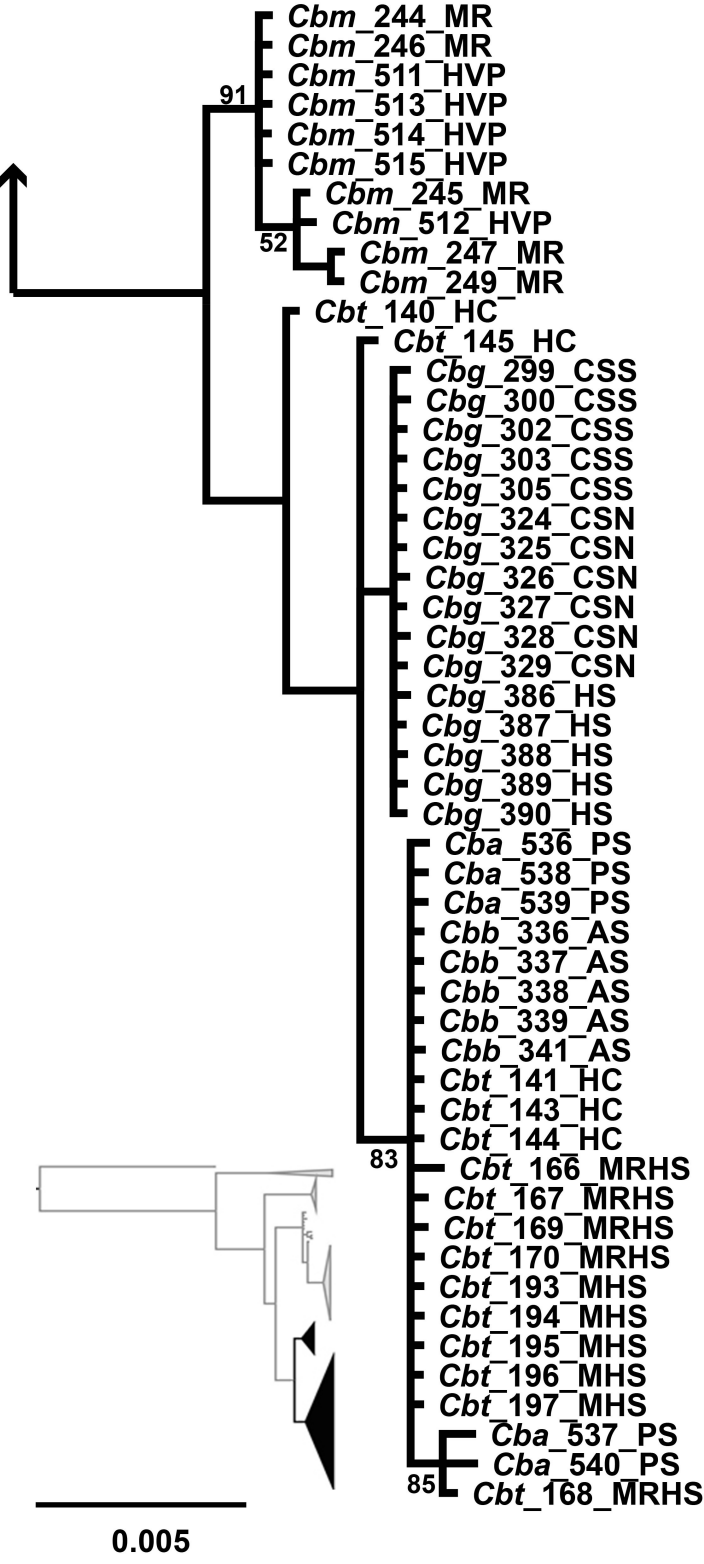
nDNA subtree of *Crenichthys baileyi*. Nuclear subtree depicting the subspecies relationships of *C*. *baileyi*, which has less resolution than the mitochondrial tree. All posterior probabilities greater than 95% unless specified on the tree.

The nuclear tree recovered fewer clades with lower posterior probabilities for the *C*. *baileyi* subspecies than the mitochondrial tree. In the nuclear tree, the populations of *C*. *b*. *moapae* were recovered as sister to all other *C*. *baileyi* with moderate posterior probability (BPP ≥ 90) and had no population structure (Fig 6). Within the clade containing *C*. *b*. *thermophilus*, *C*. *b*. *albivalis*, *C*. *b*. *baileyi*, and *C*. *b*. *grandis*, no population structure was present for the individual subspecies, however, *C*. *b*. *grandis* did form a monophyletic clade within the complex containing all the subspecies (BPP ≥ 95). This differs from the mitochondrial tree where *C*. *b*. *grandis* was recovered as sister to *C*. *b*. *baileyi*, all of which was sister to *C*. *b*. *moapae* and then to the clade containing *C*. *b*. *thermophilus* and *C*. *b*. *albivalis*.

The two trees differed in resolution and the placement of subspecies. Within *C*. *nevadae*, two lineages were discovered (the northern and southern clades) in the mitochondrial tree while only one was recovered (the southern clade) in the nuclear tree. Both of these trees did support the separation of the individuals from the northern clade from the southern clade. Within *C*. *baileyi*, four of the five subspecies were recovered forming monophyletic lineages within the mitochondrial tree while within the nuclear dataset only two were recovered, one of which (*C*. *b*. *moapae*) collapses due to low posterior probability (BPP<95). The relationships of the subspecies differed between the mitochondrial tree and the nuclear tree as well. In the mitochondrial tree, *C*. *b*. *thermophilus/albivalis* were sister to a clade containing the other subspecies, while within the nuclear tree, a clade containing *C*. *b*. *moapae* was recovered as sister to a clade containing the other subspecies. In both cases, the *C*. *b*. *moapae* individuals were separate from the other members of *C*. *baileyi* though they did not always form monophyletic clades with high posterior probability (BPP ≥ 91).

No differences were recovered between the mitochondrial and nuclear tree for *E*. *latos*, both trees recovered a monophyletic clade containing an unresolved group of all the specimens of *E*. *latos* from two populations (BPP = 100) sister to all *Crenichthys*.

### Haplotype networks

**Fig 7:**
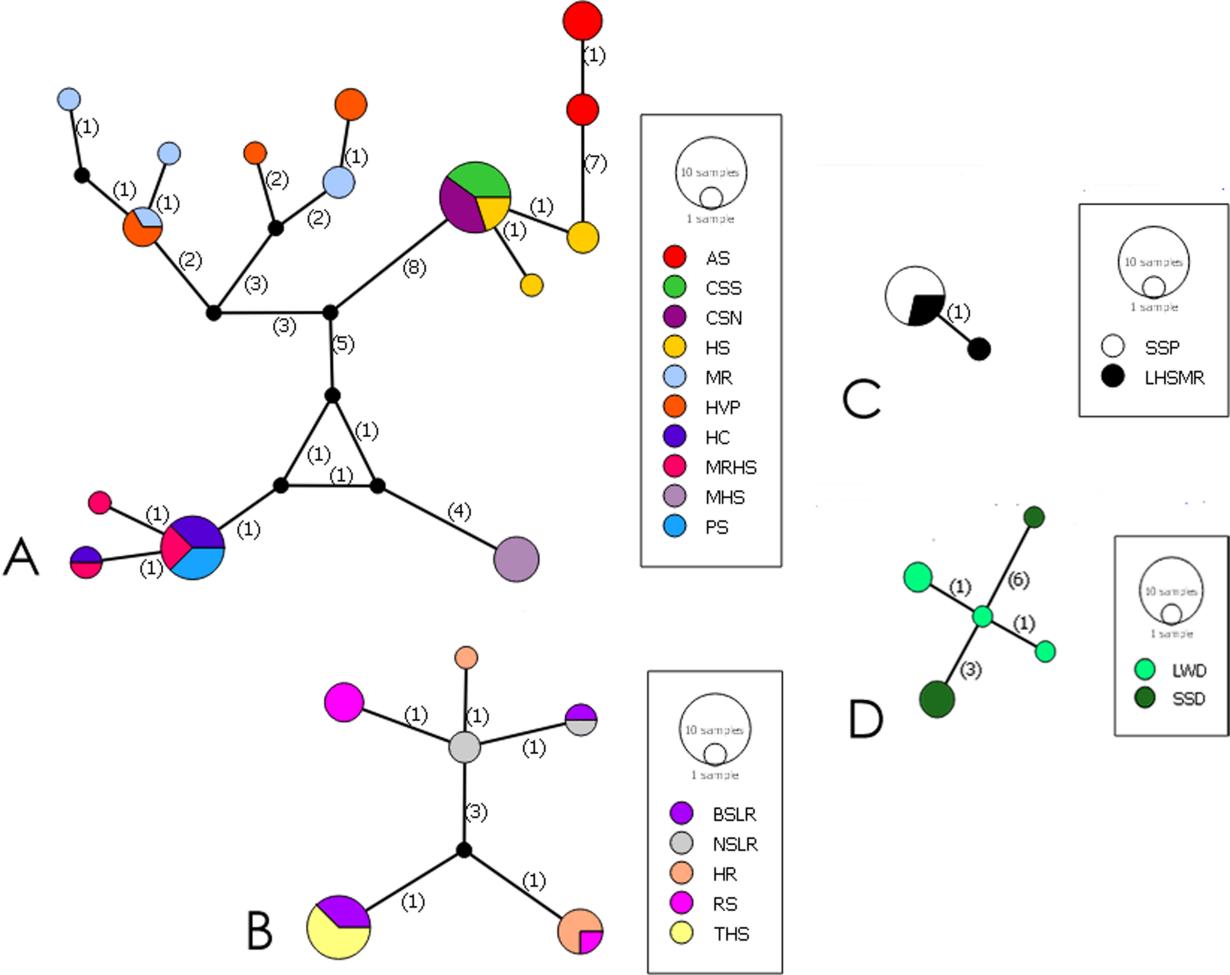
Haplotype network. 50% Majority Rule Median Joining Haplotype network based on *cytb*. Labels match Table 1 locality labels. **A-** *C*. *baileyi* complex **B-** *C*. *nevadae* southern clade **C-** *E*. *latos* network **D-** *C*. *nevadae* northern clade.

Based on the TCS 95% cutoff criterion for the cyt*b* data, the haplotype networks recovered were separated by 15 mutational steps. This separated the three-recognized species of Empetrichthyinae into individual haplotype networks (Fig 7). The unbroken network is presented in the supplemental material (S4 Fig). *Crenichthys nevadae*, inclusive of all individuals, was separated from *C*. *baileyi* by a minimum of 58 steps and *E*. *latos* by a minimum of 139 steps. *Crenichthys baileyi* was separated from *E*. *latos* by 85 steps.

Within *C*. *nevadae*, members of the northern clade were separated based on the TCS criterion into a separate haplotype network (>15 mutation steps), different from the other populations of *C*. *nevadae* by 15 mutational steps. A total of 10 different haplotypes were present within *C*. *nevadae*, four belonging to the northern clade populations, and six to the southern clade’s populations. Uncorrected (p) genetic distance (Table 2) based on cyt*b* among populations of *C*. *nevade* ranged from 0.1% to 1.8%. Distance between the northern clade populations and the southern clade populations ranged from 1.6% to 1.8%, while distance within the southern clade populations ranged from 0.1% to 0.4%. Genetic distance within *C*. *nevadae* on average was 1%.

**Table 2:** Unconnected genetic distances for both the *cytb* data and the concatenated nuclear data. Values below the middle line represent genetic distances for *cytb* data. Values above represent genetic distances for the concatenated nuclear data.

	Goodeinae	Cba	Cbb	Cbg	Cbm	Cbt	Cn	Cn_DW	El	Outgroup
Goodeinae		0.048	0.052	0.049	0.047	0.049	0.052	0.050	0.050	0.117
Cba	0.145		0.001	0.005	0.008	0.002	0.021	0.016	0.022	0.110
Cbb	0.147	0.017		0.003	0.008	0.001	0.021	0.016	0.022	0.115
Cbg	0.149	0.015	0.008		0.008	0.003	0.024	0.019	0.023	0.113
Cbm	0.147	0.013	0.016	0.015		0.008	0.018	0.013	0.020	0.110
Cbt	0.144	0.002	0.018	0.016	0.014		0.022	0.017	0.021	0.113
Cn	0.146	0.058	0.066	0.063	0.061	0.058		0.005	0.027	0.117
Cn_DW	0.146	0.058	0.066	0.064	0.062	0.058	0.017		0.022	0.113
El	0.156	0.081	0.082	0.081	0.081	0.080	0.085	0.084		0.114
Outgroup	0.187	0.188	0.192	0.193	0.190	0.188	0.194	0.189	0.188	

None of the subspecies within *C*. *baileyi* were separated by the TCS criterion into separate haplotype networks. Members of *C*. *b*. *baileyi* were separated from *C*. *b*. *grandis* by seven steps. *Crenichthys b*. *moapae* was separated from *C*. *b*. *grandis* and *C*. *b*. *thermophilus*/*C*. *b*. *albivalis* by 14.5 steps, on average. Within *C*. *baileyi*, there were a total of 15 unique haplotypes recovered. Genetic distance between populations of *C*. *baileyi* ranged from 0% to 2.0% and was on average 1.2%.

Two haplotypes were recovered for *E*. *latos* with no significant distance between either population that do not correspond to either population. Genetic distance between Empetrichthyinae and Goodeinae was between 14.5% and 15.9%. Genetic distance between Empetrichthyinae and the outgroup was between 18.7% and 20.3%.

### Species tree

**Fig 8:**
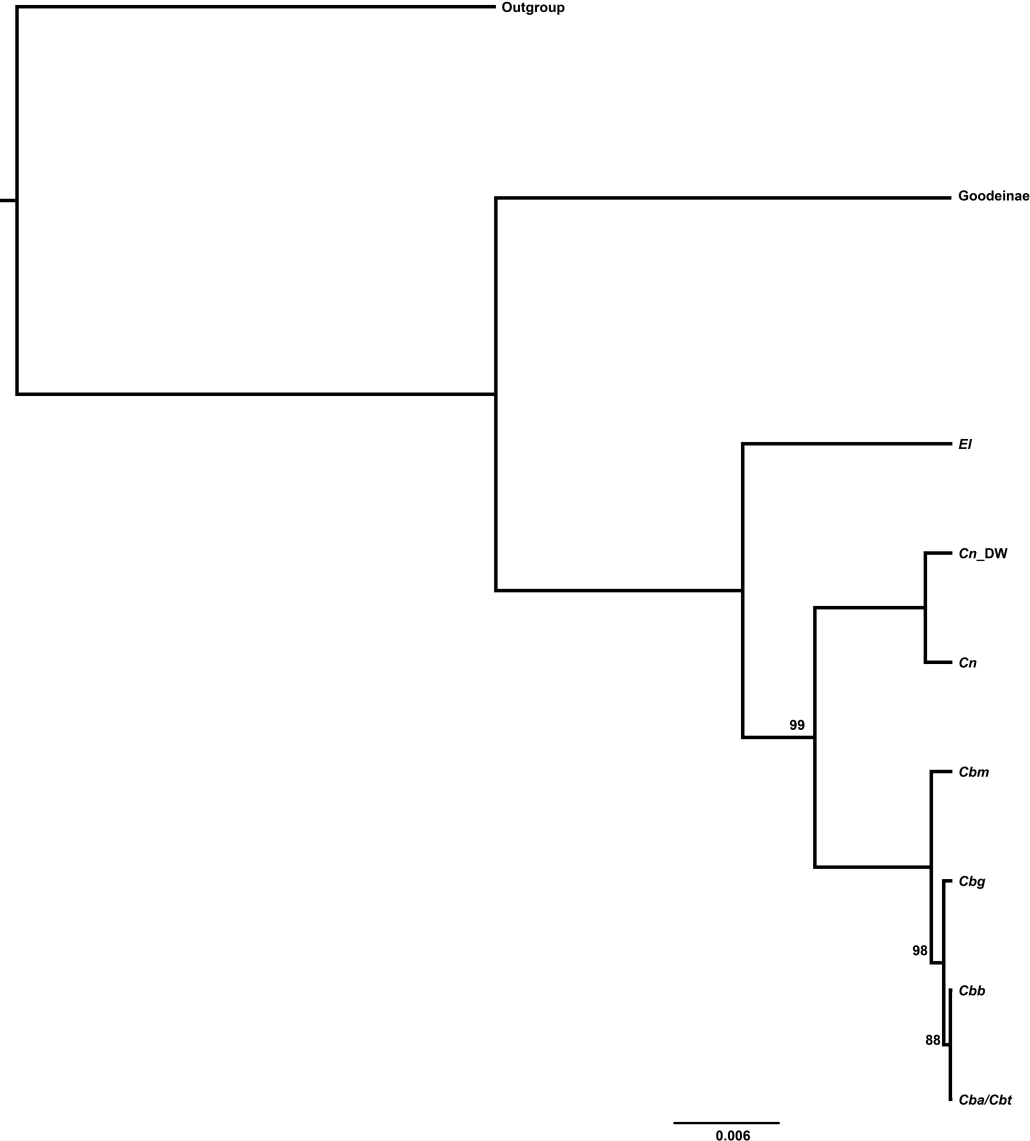
Species Tree. Phylogeny from species tree analysis based on cytb hypothesis of relationships (this study, Fig 2 or Figs 3 & 4). All posterior probabilities listed. Cn_DW represents the nouthern clade of *Crenichthys nevadae*. Other labels match the taxon labels presented in Table 1.

For the species tree analyses, the clock test showed that both P0 and S8 required strict molecular clocks while S7 required a relaxed clock. Using these models, the analysis of relationships within Empetrichthyinae using the coalescent species tree approach implemented within *BEAST produced a tree with strong posterior probability for the separate lineages within *C*. *baileyi* and *C*. *nevadae* (Fig 8). Within *C*. *baileyi*, *C*. *b*. *moapae* formed an independent lineage with high posterior probability (BPP = 100) sister to the other subspecies which formed a separate clade with lower posterior probability (BPP < 95). Members of *C*. *nevadae* from the Duckwater populations (the northern clade) also formed a separate clade with high posterior probability (BPP = 100) sister to the rest of *C*. *nevadae* (the southern clade). Trees using other species designations (nuclear lineages and morphological hypothesis) are provided in the supplementary material (S5 Fig and S6 Fig).

## Discussion

The recognition of subspecies continues to confound research in biology and limit our understanding of species relationships [2][3][5][10]. As a result, it is important to continue to critically analyze these descriptions and seek to discover the evolutionary history of species rather than clines in variation and potential “incipient species” [5]. The subspecies of *C*. *baileyi* were described based on a cline of morphological variation in the absence of phylogeny. Due to the recognition of multiple subspecies within *C*. *baileyi*, the imperiled nature of described species within Empetrichthyinae, and the disjunct distribution of each species and their populations, the subfamily requires critical analysis to better understand its evolutionary history and help inform future management practices.

This study identified multiple independent lineages within Empetricthyinae and several evolutionary lineages within *Crenichthys*. Initially, it was hypothesized that each subspecies would form independent lineages dues to their isolated distribution across multiple pools and springs within the southern portion of the Great Basin. It was also expected that cryptic species would be identified due to this disjunct distribution, however, this was not entirely the case.

While both the mitochondrial (*cytb*) and concatenated nuclear (S7, S8, P0) data sets identified multiple lineages within *Crenichthys*, they did not support the monophyly of all the described subspecies of Williams and Wilde [23]. In the mitochondrial tree, four of the five described subspecies were monophyletic (*C*. *b*. *grandis*, *C*. *b*. *baileyi*, *C*. *b*. *moapae*, and a clade containing both *C*. *b*. *thermophilus* and *albivalis*), potentially suggesting four lineages within *C*. *baileyi*. Within *C*. *nevadae*, two lineages were found rather than one (the northern and southern clades), suggesting a possible as yet undescribed species. The nuclear dataset presented a more conservative estimation of lineages within *Crenichthys*, with two lineages within *C*. *baileyi* (*C*. *b*. *moapae* sister to a mixed comb of all other *C*. *baileyi*) and again recovering the split within *C*. *nevadae* (the northern and southern clades).

In both datasets, only one lineage was found for *E*. *latos*, which was expected as *E*. *latos* is no longer found within its home range in the Pahrump Valley, and is currently maintained in stock ponds outside of its native range within the Great Basin. The difference in phylogenetic estimation between the mitochondrial and nuclear datasets is expected due to the higher rate of mutation within the mitochondrial genome and the greater variation present versus the slower mutation rate of nuclear DNA [53][54].

The lineages described could represent potential management units or undescribed species within the genus, and are further supported by the geographic separation of each lineage. Taking the more conservative approach and focusing on the nuclear dataset, *C*. *b*. *moapae* is the most geographically disjunct member of *C*. *baileyi*, more than 80 kilometers away from the most geographically proximate populations in the Pahranagat Valley. *Crenichthys b*. *moapae* are separated by Pahranagat Valley and Sheep Range in the west, while to the east the Delmar and Meadow Valley Mountains represent a geographic barrier. There is presently no physical connection between the populations. A similar case is present for the northern and southern clades within *C*. *nevadae*, with members of the northern clade separated from the southern clade by Pancake Range with over 40 kilometers between the northern clade and the other spring systems containing the southern clade individuals.

Based on this information, the separation of these lineages represents species boundaries and suggest that species level diversity within *Crenichthys* is different than previously described. This is supported by the *BEAST species tree analysis which recovers the separation of *C*. *b*. *moapae* from the other members of *C*. *baileyi* and the separation of northern clade populations of *C*. *nevadae* from southern clade populations of *C*. *nevadae* with high posterior probability (BPP = 100). This result was recovered by all the species tree analyses, regardless of the starting hypotheses (Supplementary data, S5 Fig and S6 Fig). This method is based on the coalescent theory, an approach that models genetic drift and population size in combination with analyzing the gene trees within data separately to develop a species tree, rather than as one partitioned locus. Species trees have been used in a variety of studies to identify and separate multiple species where diversity is cryptic and resources are limited [22][55][56]. Posterior probability for the other subspecies of *C*. *baileyi* each forming an independent lineage was low (BPP = 88) and suggests they correspond to the same species based on the nuclear data, though this does not suggest that they are not genetically distinct enough to not be considered possible management units or ESUs. However, due to the low-resolution among populations in this study, a more in-depth population genetics study in the future is necessary to identify management units at the population level. It is worth noting that sequences for *C*. *b*. *thermophilus* and *C*. *b*. *albivalis* were identical in both the mitochondrial and nuclear data sets, suggesting that these populations likely represent the same taxon.

Genetic distances between the discovered lineages were low within *Crenichthys* based on *cytb*, however, this may be a trait common across the family Goodeidae and a result of the recent estimated divergence time for *Crenichthys* [57]. This study does not advocate the sole use of genetic distances as a method to recognized species, however, for comparative purposes, genetic distances between *C*. *baileyi* spp. and *C*. *b*. *moapae* ranged between 1.3% and 1.7%, which is low, but still coincides with distances used for currently described species in the family, including species from their sister subfamily Goodeinae [57][58]. A similar range is present for the distance between *C*. *nevadae* and the Duckwater populations at 1.6–1.8%. As a result, the haplotype networks did not offer as much support for separation within *C*. *baileyi*, due to the low genetic distance within the group as a whole. Within the haplotype network analysis, networks were separated using a 95% cutoff criterion within TCS (15 steps). Based on this criterion, the separation of individual subspecies or populations within *C*. *baileyi* was not recovered, though *C*. *b*. *moapae* was the closest to separation at 14.5 mutational steps from the other most closely related subspecies (Fig 7). Within *C*. *nevadae* however, the separation of the northern clade populations from the southern clade populations of *C*. *nevadae* was supported by the TCS cutoff criterion at 15 mutational steps from the other members of *C*. *nevadae*.

## Conclusion

Using multiple loci, evolutionary lineages within Empetrichthyinae were identified to re-analyze the relationships of the described subspecies within the subfamily to accomplish two objectives–generating phylogenies for Empetrichthyinae representing individuals from across their entire range and re-evaluating their relationships based on these phylogenies, which were both completed. As a result, this study identified cryptic diversity within the group and invalidated some of the subspecies descriptions based on the Evolutionary Species Concept. Based on the mitochondrial phylogeny, there are four lineages within *C*. *baileyi*, while based on the nuclear loci there are two lineages. Therefore, the most conservative conclusion would suggest there are two species within *C*. *baileyi*. As for *C*. *nevadae*, both the mitochondrial and nuclear data suggest that there are two lineages as well, implying there may be two species where only one is described. These results are supported by the species tree analysis and the geographic separation of these lineages within both *C*. *baileyi* and *C*. *nevadae*. The separation of the northern and southern clade of *C*. *nevadae* is further supported by the haplotype network analysis.

These results represent the first steps toward a better understanding of the relationships and taxonomic status of the populations within Empetrichthyinae and suggest that more work within the subfamily is necessary. Subspecies continue to be an issue that confound our understanding of evolutionary history, but thankfully, many have already realized this thanks to past and new species concepts, and many subspecies continue to be re-evaluated ebased on these concepts [5][17][18][59][60][61]. Considering the results of this study, a comprehensive morphologically based taxonomic revision of *Crenichthys* is necessary to compliment these results before describing these lineages as new species. Future population genetic studies of *Crenichthys* are needed to obtain a fine-scale picture of the genetic diversity within the subfamily to inform conservation practices and identify management units.

## Supporting information

S1 Fig Gene tree of P0 intron 1. Fifty-percent majority rule gene tree of P0 intron 1. Labels match Table 1.
(TIF)

S2 Fig Gene tree of S7 intron 1. Fifty-percent majority rule gene tree of S7 intron 1. Labels match Table 1.
(TIF)

S3 Fig Gene tree of S8 intron 1. Fifty-percent majority rule gene tree of S8 intron 4. Labels match Table 1.
(TIF)

S4 Fig Hapolotype network TCS cutoff. 50% Majority Rule Median Joining Haplotype network based on *cytb* before separation based on TCS 95% cutoff criterion. Labels match Table 1 locality labels.
(TIF)

S5 Fig Species tree based on morphological designations. Phylogeny from species tree analysis based on morphological hypothesis of relationships (Williams & Wilde, 1981). All posterior probabilities listed. Cn_DW represents the northern clade of *Crenichthys nevadae*. Other labels match the taxon labels presented in Table 1.
(TIF)

S6 Fig Species tree based on nuclear sequence designations. Phylogeny from species tree analysis based on nuclear hypothesis of relationships (Fig 2 or Figs 5 & 6). All posterior probabilities listed. Cn_DW represents the northern clade of *Crenichthys nevadae*. Other labels match the taxon labels presented in Table 1.
(TIF)
