# Supplementary figures and images for: Let's jump in: A phylogenetic study of the great basin springfishes and poolfishes, Crenichthys and Empetrichthys (Cyprinodontiformes: Goodeidae)
Source: PLoS One. 2017 Oct 27;12(10):e0185425. doi: 10.1371/journal.pone.0185425 (PMC5659628; doi:10.1371/journal.pone.0185425)

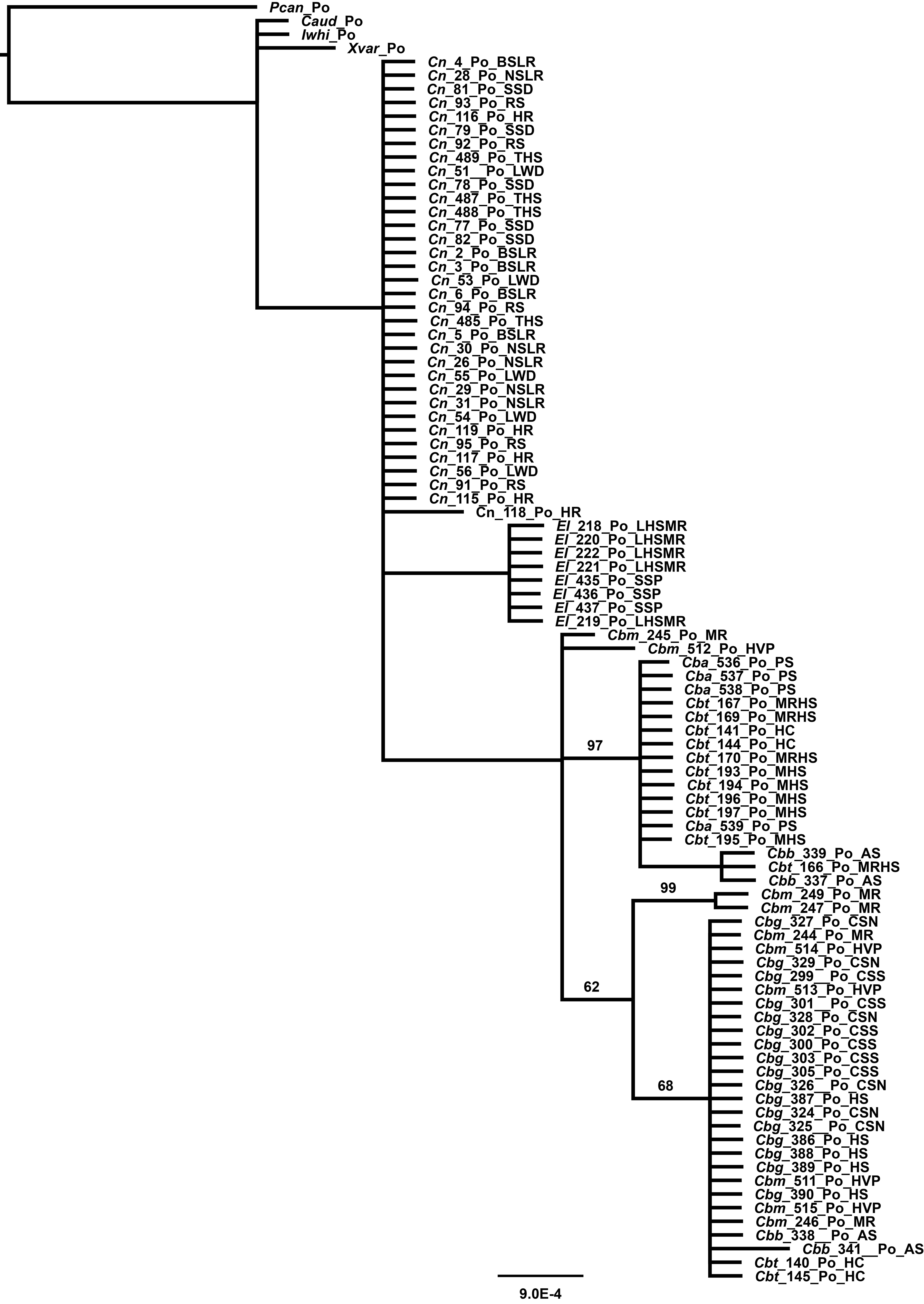

Supplement: S1 Fig — Fifty-percent majority rule gene tree of P0 intron 1. Labels match Table 1. (TIF) [file pone.0185425.s001.tif]

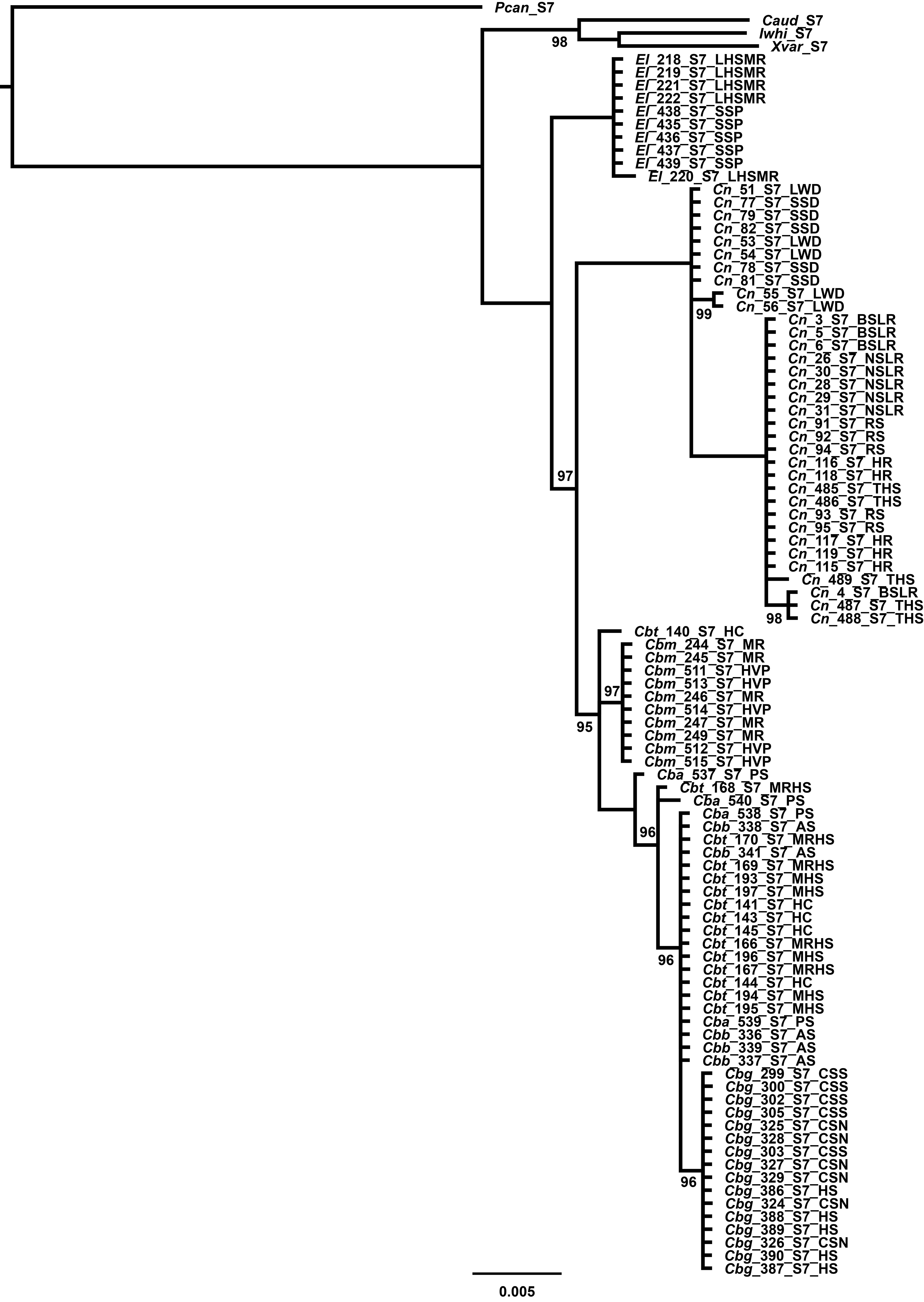

Supplement: S2 Fig — Fifty-percent majority rule gene tree of S7 intron 1. Labels match Table 1. (TIF) [file pone.0185425.s002.tif]

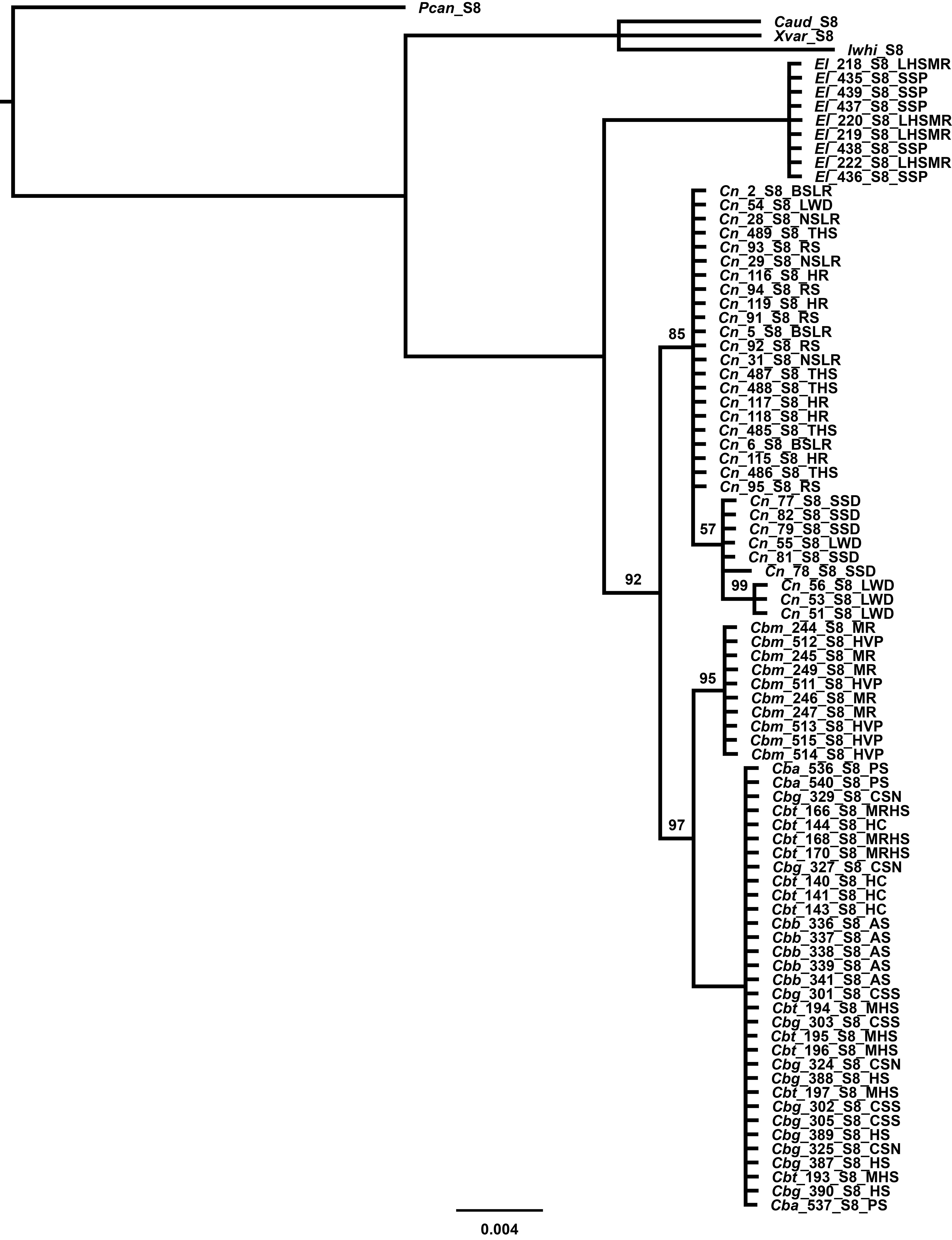

Supplement: S3 Fig — Fifty-percent majority rule gene tree of S8 intron 4. Labels match Table 1. (TIF) [file pone.0185425.s003.tif]

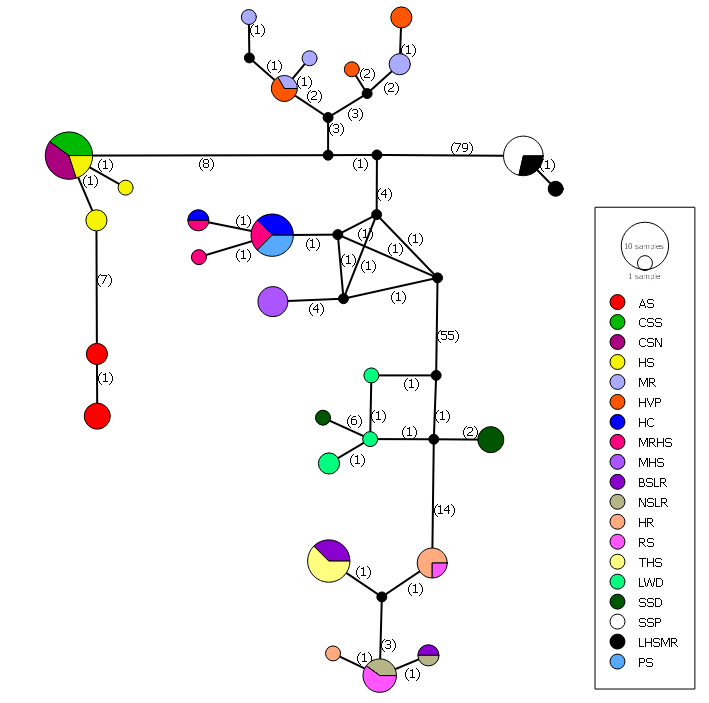

Supplement: S4 Fig — 50% Majority Rule Median Joining Haplotype network based on cytb before separation based on TCS 95% cutoff criterion. Labels match Table 1 locality labels. (TIF) [file pone.0185425.s004.tif]
